# Flexible and Stretchable Microneedle Electrode Arrays by Soft Lithography for Continuous Monitoring of Glucose

**DOI:** 10.3390/bios15090576

**Published:** 2025-09-02

**Authors:** Yong-Ho Choi, Honglin Piao, Jia Lee, Jaehyun Kim, Heon-Jin Choi, Dahl-Young Khang

**Affiliations:** Department of Materials Science and Engineering, Yonsei University, Seoul 03722, Republic of Korea; yhyhyh825@naver.com (Y.-H.C.); hongrim@yonsei.ac.kr (H.P.); jia.lee@yonsei.ac.kr (J.L.); kimjae5020@yonsei.ac.kr (J.K.)

**Keywords:** continuous glucose monitoring, microneedle electrode arrays, soft lithography, impedance spectroscopy

## Abstract

Continuous monitoring of glucose (CGM) level is of utmost importance to diabetic patients, especially with no or minimal pain. Microneedle arrays with desired electrode patterns have been fabricated by soft lithographic molding, and the patterned electrodes were formed via shadow evaporation through a shadow mask that was made from a modified molding technique. With immobilization of glucose oxidase (GOx), the microneedle electrode arrays (MEAs) have been successfully employed for the in vitro CGM using impedance spectroscopy. The fabricated MEAs could monitor the varying glucose level continuously for up to ~10 days. Similar processes have been applied for the fabrication of stretchable MEAs, which can conform to complex curvilinear surfaces. The simple and low-cost fabrication of MEAs, either in flexible or stretchable forms, may find various applications in wearable health monitoring techniques.

## 1. Introduction

Microneedles are defined as micron-sized needles, with their height ranging from 25 to 2000 μm. They are made of various materials, such as metals, ceramics, and polymers, and have different shapes for specific applications, such as solid, hollow, coated, or dissolvable [1,2]. Usually, the microneedles are used in an arrayed format, called microneedle arrays (MNA), which can be fabricated by using various processing methods such as lithography or micro-molding. They have a variety of applications, ranging from drug/vaccine delivery, patient monitoring, and disease diagnosis to cosmetic applications. One of the unique advantages of the MNA is its painless or minimally invasive nature [3,4].

On the other hand, microneedle electrode arrays (MEAs) are an extension of MNAs that are coated with conductive materials by various deposition techniques [5]. The addition of electrical conduction, i.e., the formation of microneedle electrodes, enables various applications using electrical signals. Therefore, the applications of MEAs include the monitoring of bio-signals, such as electrocardiography (ECG), electromyography (EMG), and electroencephalography (EEG) [6]. The MEAs can also be used for neural interfaces with the capability of neural recording and neural stimulation [7]. These bio-signals are vital for understanding the pathological and physiological conditions of patients.

In the meanwhile, diabetes, a chronic metabolic disorder due to impaired insulin secretion or action, is one of the significant public health concerns worldwide [8]. In general, the diabetic patients experience an abnormal fluctuation of glucose levels in the body. The glucose level in the body should be maintained in a specific range; otherwise, diabetes may lead to severe complications such as high blood pressure, strokes, retinal damage, neuropathies, kidney failure, skin ulcers, and cardiovascular diseases. Thus, continuous monitoring of the glucose level, with concomitant insulin medication, is the only solution for diabetes. The glucose level has been measured by an invasive finger-prick method, puncturing the finger with a lancet followed by analyzing the blood glucose level with a glucose meter, which is inconvenient and painful to patients. Moreover, it does not provide complete glucose level dynamics even with such frequent finger pricking [9]. Therefore, it is essential to develop a method to monitor the glucose level continuously by attaching a device to the human body in a wearable form and providing glucose readings every 1~5 min along with glucose trends and alarms for hypo and hyperglycemic conditions [10]. Further, the continuous glucose monitor (CGM) can be connected to an insulin injection pump to adjust delivery in response to the monitor readings.

There have been numerous sensing methods for CGM [11,12,13,14,15,16,17,18], which can be roughly classified into non-invasive, minimally invasive, and invasive ones. Non-invasive CGMs are mostly related to the use of optical or spectroscopic approaches and some transdermal methods such as impedance spectroscopy and reverse iontophoresis; invasive CGMs include subcutaneous enzymatic sensors and are now available in the market. Minimally invasive CGMs are based on the use of microneedles, where the needles in hollow or microporous form extract interstitial fluid for further in vitro glucose level determination.

In this work, we have demonstrated the facile fabrication of MEAs with no need for expensive lithography processes. The method developed here exploits proper control of interfacial adhesion between materials involved, in addition to transparent and conformable polymeric shadow masks that are made by a modified molding technique [19,20]. Specifically, electrode materials (Au/Ti) were sequentially deposited, through the transparent shadow mask, onto a hole-patterned elastomeric polydimethylsiloxane (PDMS) stamp, which completes the formation of patterned MEAs. Upon peeling off the poured/cured polymeric material directly yields MEAs having conductive materials on the whole 3-dimensional parts of the sample, together with 2-dimensional patterned interconnections and contact pads at the same time. The optical transparency of the polymeric shadow mask enables easy and accurate alignment onto the patterned elastomeric substrate manually. Also, the shape of microneedles can be easily manipulated, from simple cylinders to cone-like shapes, by exploiting the non-conformal coating of viscous liquid (polyimide varnish in this work) into the holes on elastomeric PDMS.

Depending on the needle shape, the skin penetration mechanism differs: mode I crack opening for cone-like microneedles and mode II shearing with simple cylinder-shaped needles [21,22]. The prepared MEAs have been applied for CGM using impedance spectroscopy. CGMs based on impedance spectroscopy do not involve any specific chemistry [23], thus are vulnerable to many parameters that are not easy to control, such as sweat, temperature, and intimate contact between sensor and skin, to name a few. On the contrary, we have immobilized glucose oxidase (GOx) on MEAs, where the chemical reaction between GOx and glucose, including consecutive reactions, leads to the change in impedance that can be measured by MEAs. In general, enzyme-functionalized MEAs have been applied for CGM using electrochemical measurements [24]. The impedance spectroscopy with enzyme-immobilized MEAs demonstrated in this work, however, has shown very promising results, in that the method is very sensitive and accurate to glucose level thanks to the redox chemistry involved. Further, the impedance-based CGM with enzyme-functionalized MEAs was found to be reliable and robust for long-term CGM, up to ~10 days with skin simulant. Overall, the simple and easy fabrication of MEAs based on the molding technique has successfully been demonstrated, and their application to CGM with GOx immobilization has shown promising results for minimally invasive CGM.

## 2. Materials and Methods

### 2.1. Materials

Glucose oxidase (GOx), D-(+)-glucose, N-hydroxysuccinimide (NHS), 3-mercaptopropionic acid (MPA), and 1-ethyl-3-(3-dimethylaminopropyl)carbodiimide hydrochloride (EDC) were purchased from Merck or Sigma (Seoul, Republic of Korea), which were used without any further purification. The pH of phosphate buffer solution (PBS) was adjusted by potassium dihydrogen phosphate or disodium hydrogen phosphate dilute solution. Polydimethylsiloxane (PDMS) was purchased from Dow (Sylgard 184, Midland, MI, USA) and used for the fabrication of elastomeric MEAs and for stamps in MEA fabrication. Thermoset, UV-curable polyurethane acrylates (PUA; MCnet, Seoul, Republic of Korea) were used as a substrate for MEAs (PUM-W6070) and a freestanding shadow mask (PUM-3117) for patterned electrode deposition. Trichloro(1H,1H,2H,2H-perfluorooctyl)silane from Aldrich (Seoul, Republic of Korea) was used to make the PDMS surface non-stick for consecutive replication. Polyimide (PI) varnish was kindly supplied by PI Advanced Materials (Seoul, Republic of Korea) and used for the shape control of microneedles. Eco-flex (Eco-flex 0050, Macungie, PA, USA) elastomer was employed as skin simulation for the penetration test. Swine skin, micropig Franz cell membrane (MFCM; UMC Science, Seoul, Republic of Korea), which has a similar structure to human skin, was also used for the penetration test and glucose monitoring. A commercial animal blood (sheep blood, Hardy Diagnostics, Santa Maria, CA, USA) was used for glucose sensing. For this, a specific amount of glucose was added into the animal blood for the test.

### 2.2. MEAs Fabrication and Microneedle Shape Control

**Figure 1:**
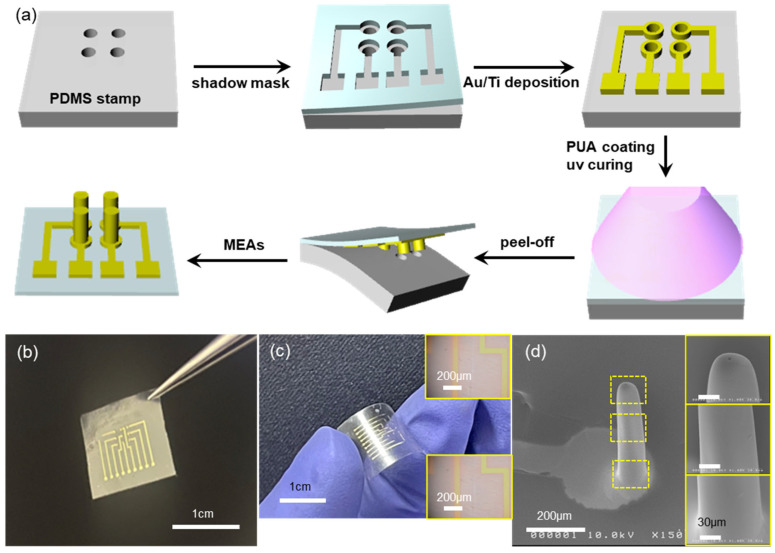
Fabrication of MEAs via simple and low-cost processes. (**a**) Schematic drawings for the MEAs fabrication steps. (**b**) Photo image of the fabricated MEAs on flexible polymer substrate. (**c**) Photo image showing the bent MEA substrate, with inset OM images of metal electrode before (**top**) and after (**bottom**) the bending deformation, respectively. (**d**) SEM image of a needle electrode covered with patterned Au/Ti layers. The inset SEM images correspond to the regions enclosed by dotted yellow rectangles from top to bottom, respectively.

The MEAs were fabricated based on the replica molding technique. A master mold was a stainless steel plate (0.8 mm in thickness) with laser-drilled through-holes in 3 × 3 array format. After blocking one side of the steel plate, the PDMS stamp was replicated by mixing base resin and curing agent in a weight ratio of 10:1, de-aired, and thermally cured at 70 °C for 3 h. The replicated PDMS stamp now has protruding needle arrays, which should be inverted into the opposite tone for the MEA fabrication. For the inversion, the needle-arrayed PDMS was O_2_-plasma treated in home-made equipment (30 W, 25 sccm O_2_, 3 min) and treated with fluorinated self-assembled monolayer (F-SAM) to reduce the interfacial adhesion. Then, another round of replica molding with PDMS was carried out onto the surface-treated, needle-arrayed PDMS stamp, which yields a PDMS stamp having hole arrays. The fabrication of MEAs on the hole-arrayed PDMS stamp will be explained in Figure 1a in greater detail.

For the control of microneedle shape, PI varnish was spun onto the hole-arrayed PDMS stamp. Prior to the coating, the PDMS stamp was O_2_ plasma treated to enhance the wetting of PI varnish. Due to the viscous nature of the PI varnish, together with the high aspect ratio of the hole structure (hole depth ~800 μm, hole diameter ~200 μm, aspect ratio of 4), the varnish coating is not conformal and uniform along the hole. To be specific, the PI accumulates at the hole bottom, while the hole entrance has a rather thin and uniform coating layer. Another round of replica molding was done with the PI varnish-coated PDMS stamp, which leads to a sharpened microneedle top with a wide bottom. Repetition of the varnish coating and replication makes the microneedle shape change from a simple cylinder to cone-like shapes.

### 2.3. MEAs Penetration Test

The penetration test was performed on swine skin and eco-flex elastomer as simulants to human skin. The MEAs were manually pressed onto the skin simulants. The skin simulants after the penetration test were cut to image the cross-section, which enabled us to discriminate the crack mode during skin penetration, depending on the shape of microneedles. In addition, the top surface of the skin simulant was imaged, which again tells us the mode of crack propagation. Although the penetration test should involve more detailed mechanical characterization, such as penetration force and level of pain or discomfort experienced by the patient, in this work we primarily focus on the crack mode analysis depending on the microneedle shape.

### 2.4. Glucose Sensor Fabrication and Measurement

The processed MEAs were thoroughly rinsed with de-ionized (DI) water before GOx immobilization. Then the MEAs were put into a 1:3 DI/ethanol solution containing 20 mM MPA for 24 h, which forms a self-assembled monolayer of MPA through Au-S bonding. The MPS-modified MEAs were repeatedly washed with ethanol, dried in an Ar stream, and activated in PBS (pH = 5.5) solution containing 0.002 M EDC and 0.005 M NHS for an hour. Again, the MEAs were rinsed with the copious volume of PBS (pH = 5.5) and then immediately placed in PBS (pH = 5.5) containing 500 μg/mL of GOx enzyme for 1.5 h. This completes the Au-MPA-GOx immobilization, which can be used for glucose monitoring. The same processes were done on Au film deposited on a cleaned Si substrate to measure the change in surface morphologies by AFM before/after the GOx immobilization.

The GOx-immobilized MEAs were integrated onto the FPCB pad with Au wire bonding for glucose monitoring. The MFCM was fully immersed into a glucose solution (concentration ranges from 50 mg/mL to 200 mg/dL) in PBS for more than 12 h. Then, the MFCM soaked with glucose solution was loaded onto the MEAs on the FPCB substrate. The impedance was measured by applying sinusoidal AC current in the frequency ranging from 100 Hz to 1 MHz using an impedance analyzer (E4990A; Keysight, Santa Rosa, CA, USA). Separately, glucose concentration was measured by a commercial glucose meter (CareSens II Plus; i-SENS, Inc., Seoul, Republic of Korea) for comparison. Long-term continuous glucose monitoring (CGM) was also carried out in a similar way. Here, the glucose concentration was varied by applying different concentrations of glucose solution onto the MFCM. The MEA sensor with MFCM was maintained wet in a Petri dish saturated with PBS so as not to dry the MFCM. Once the MFCM starts to dry, the impedance value does start to increase, too. This increase in impedance was used to find optimum timing for applying glucose solutions having different concentrations. It should be noted that once the MFCM was fully dried during long-term CGM, the sensor was degraded permanently: even under the application of glucose solution having different concentrations, the sensor did not change its response at all. Then, the sensor was discarded, and new MEA sensors with fresh MFCM soaked with glucose solution were replaced, and the measurement started again.

For stretchable MEAs made of elastomeric PDMS, it is impossible to directly wire bond between contact pads on PDMS and those on FPCB. This is due to the extremely soft nature of PDMS: once any mechanical deformation, such as unintentional touching or probing with sharp tips, etc., occurs, the metal electrode on PDMS completely shatters apart, making the whole sample useless. Therefore, special steps were taken to make a sort of electrical extension the contact pads were mutually connected with thin Au wires, with the end of the wire fixed with Ag paste on the stretchable MEAs.

## 3. Results

There have been a variety of different fabrication techniques for microneedle arrays (MNAs) [1,2,3,4], which can be easily extended to the fabrication of microneedle electrode arrays (MEAs). This is mainly due to the fact that MEAs can be simply prepared from MNAs by depositing electrode materials (metal thin films, in general) onto the MNAs using various deposition techniques such as sputtering, evaporation, and so on. In this work, we combined the two processes, MNA fabrication and patterned electrode deposition, into a single step, without using any costly processes such as lithography. The fabrication process starts with the hole-patterned elastomeric PDMS stamp, as shown in the first step of Figure 1a, where the patterned PDMS stamp was replica-molded from a laser-drilled stainless steel plate (Supporting Information, Figure S1), which is quick and cost-effective, too. The diameter and depth of holes on the PDMS stamp were 200 μm and 800 μm, respectively, with mutual spacing of 1 mm. Then, a proper shadow mask made of flexible polymer (will be discussed later) was manually aligned onto the patterned PDMS stamp (2nd step in Figure 1a). The manual alignment was enabled by the transparent nature of the shadow mask and by the rather large (hundreds of μm or larger) feature size involved. The sample was then evaporated with thin metal films, Au(80 nm)/Ti(10 nm) in the present work, using a home-built electron-beam evaporator. Upon peeling off the shadow mask, the PDMS stamp now has patterned electrodes on it (3rd step in Figure 1a). Drop-casting and UV-curing of curable polymer precursor (PUM-W6070, McNET) forms the MEAs with a thin (~80 μm) overcoat layer as shown in the 5th step of Figure 1a. It should be noted here that the depth of holes on the PDMS stamp is ~800 μm deep, which means that simple casting of a viscous precursor solution would not guarantee the complete filling of such deep holes. Thus, a vacuum was applied to remove trapped air in the holes and facilitate the filling of viscous polymer precursor. Upon peeling off the cured polymer, it has three-dimensional microneedle arrays that are fully covered with metal thin film (i.e., MEAs), in addition to proper two-dimensional interconnection lines and contact pads at one end of the sample, as shown in the last step in Figure 1a. The fabrication of MEAs shown in Figure 1a does not need any costly lithographic processes such as photoresist coating, exposure with photomask, and wet chemical development; instead, the formation of microneedle arrays and the 3D/2D patterned electrodes was carried out in sequential steps of patterned evaporation through a shadow mask, prepolymer casting and curing, and peel-off.

Figure 1b shows a photo image of the fabricated MEAs. There are nine needle electrodes arrayed in a 3 × 3 pattern. Depending on the amount of prepolymer dispensed onto the electrode-patterned PDMS stamp, the MEAs can be very flexible, as shown in Figure 1c. The inset OM images of Figure 1c show the metal interconnection lines before (top) and after (bottom) the bending deformation. There are no noticeable cracks in the metal electrodes upon bending deformation. The flexible nature of our MEAs may be beneficial in applications where the MEAs should be conformed onto non-flat curved surfaces [25,26]. Figure 1d shows the SEM image of a microneedle electrode: the microneedle (200 μm in diameter and 800 μm in height, same as the hole dimension on the PDMS stamp) is conformally coated with metal (Ti/Au) electrode and the electrode is patterned very well. The inset SEM images show the magnified view of the needle surface, where the metal film shows no noticeable cracks or any defects.

The success of our process, the patterned electrode deposition with shadow mask and peel-off of molded MEAs with metal film intact, lies in the delicate control of adhesion between materials. For the metal film deposited on the PDMS stamp, the interfacial adhesion should be weak for the following peel-off. At the same time, the interfacial adhesion between the metal film and the cured polymer should be strong enough for the successful peel-off. For this, we have deposited Au onto the patterned PDMS stamp, followed by Ti deposition. In general, Au is inert and has weak adhesion with most materials such as semiconductors, insulators, or polymers. On the contrary, the Ti layer is often used as an adhesion layer due to its better adhesion to those materials. Therefore, the deposited Au/Ti metal films have relatively weak adhesion with the underlying PDMS stamp (note that PDMS also has very weak adhesion with other materials in its pristine form) because the Au is in direct contact with PDMS. On the other hand, the Ti makes direct contact with the cured polymer (PUA), leading to quite strong adhesion. In all, the delicate control of interfacial adhesion between materials (PDMS-Au-Ti-PUA) has enabled simple and facile fabrication of MEAs, as shown in Figure 1. Otherwise, Ti/Au, instead of Au/Ti, has led to the failure of the MEA fabrications (Figure S2). The process can be applied to MEAs having other dimensions, but it cannot be used for MEAs having re-entrant shape (i.e., a wider top with a narrower bottom) (Figure S3).

**Figure 2:**
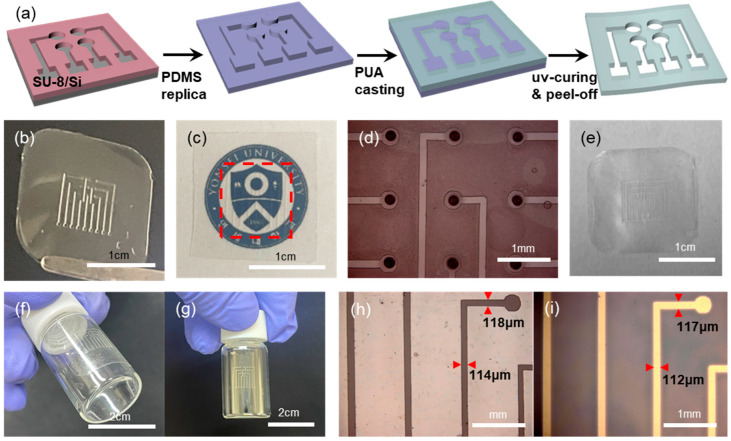
Flexible, transparent shadow mask by modified replica molding. (**a**) Schematic illustration of the fabrication steps for polymeric shadow mask by modified replica molding technique. (**b**) Photo image of shadow mask. (**c**) Photo image of the logo of Yonsei University overlaid with the transparent shadow mask (denoted by red dotted box). (**d**) Transparent shadow mask aligned onto a patterned PDMS stamp. (**e**) Photo image of shadow mask after 3 times of deposition/cleaning cycles. (**f**,**g**) Shadow mask conformed onto a curved surface of a glass vial (**f**), and the shadow-evaporated Au patterns on the vial (**g**), respectively. (**h**,**i**) OM images of interconnection lines on a shadow mask (**h**) and the shadow-evaporated Au lines (**i**), respectively.

Another enabling element in our approach is the polymeric shadow mask. Shown in Figure 2a is the schematic illustration of the shadow mask fabrication. A PDMS stamp having protruding features is replicated from a master having patterned negative photoresist (SU-8) on a cleaned Si substrate. The thickness of the SU-8 photoresist is in the range of ~50 μm, which determines the thickness of the final shadow mask. Upon the PDMS stamp, an UV-curable prepolymer (PUM-3117) is dropped and spread with a glass pipette manually. Before the UV-curing of the prepolymer, the excess prepolymer on top of the pattern features of the PDMS is removed by mechanical scraping to expose the top surface of the patterns on the PDMS stamp. Following UV-curing and peel-off leads to the freestanding shadow mask film [19,20]. Figure 2b presents a photo image of such a polymeric shadow mask. It can be noted that the shadow mask is optically transparent, as shown in Figure 2c. The existence of a shadow mask on top of our university logo (red dotted rectangle in Figure 2c) is nearly indiscernible. This optical transparency of the shadow mask has facilitated the manual alignment onto the hole-patterned PDMS stamp, as shown in Figure 2d. Even with manual handling, the alignment is quite good, due largely to the fact that the feature size involved is in the hundreds of microns range. Another advantage of our shadow mask is its recyclability. After the metal deposition with such a transparent shadow mask, the mask becomes opaque due to the deposited metal films. Then the mask with the deposited metal films can easily be cleaned again by etch-removal of the metals, leading to the transparent shadow mask again: shown in Figure 2e is the photo image of a shadow mask after 3 cycles of deposition/cleaning. In principle, the mask can be recycled indefinitely.

Furthermore, the shadow mask is thin and flexible, which enables conformal contact with curved surfaces. The transparent and flexible shadow mask was conformally attached onto a glass vial in Figure 2f. After the deposition of metal, the glass vial surface has a patterned electrode, Figure 2g, upon the removal of the shadow mask. Also, the flexible and conformable nature of our shadow mask has led to the pattern transfer with high fidelity. Shown in Figure 2h,i are the OM images of line-shaped openings on a shadow mask (Figure 2h) and the deposited metal lines on a substrate surface (Figure 2i), respectively. There is negligible variation in the linewidths between features on the mask and those on the deposition surface. This is largely due to the conformal contact of the shadow mask on the deposition surface, together with the near-vertical sidewall of the pattern features on the shadow mask (Figure S4).

**Figure 3:**
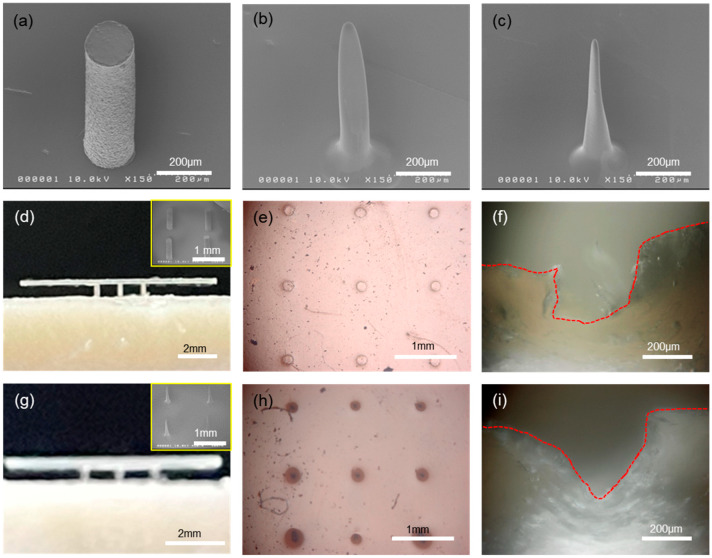
Microneedle shape control and penetration test. (**a**–**c**) SEM images of microneedles having different shapes from simple cylinder to cone-like geometry. (**d**–**f**) Penetration test of cylinder-shaped microneedle arrays into eco-flex (**e**) and porcine skin (**d**,**f**), respectively. (**g**–**i**) Penetration test of cone-shaped microneedle arrays into eco-flex (**h**) and porcine skin (**g**,**i**), respectively. Inset SEM images in (**d**,**g**) show the respective microneedle arrays used.

In general, the shape of the microneedle in MNAs or MEAs is known to be a very important parameter in terms of penetration mechanics into skin [17,22,27,28]. In our process, the shape of the microneedle can be modified from a simple cylinder to a cone-like shape, as shown in Figure 3a,c. Starting with a simple cylinder-shaped microneedle, the tip of the needle can be reduced in radius of curvature. This was made possible by spin-coating the PDMS stamp having hole patterns with a polymer solution (Figure S5). When the cylindrical hole is spun with polyimide (PI) varnish solution, the coating is not uniform everywhere; instead, the bottom region of the hole gets thicker compared to the thickness around the sidewall. That is, there is a thickness gradient from a thicker bottom to a thinner top along the sidewall, due primarily to the confined geometry. Thus, the initial cylindrical hole changes into a wider top with a narrower bottom in shape, which leads to the needle shape with a sharp tip and wide bottom upon replication with PUA, as shown in Figure 3b. Using this microneedle as a master mold, another PDMS stamp can be replicated, and now the holes in the PDMS stamp take the microneedle shape of Figure 3b with an upside-down configuration. The same process, spinning of PI solution into this modified PDMS stamp and replication of microneedles, can be repeated, which leads to much narrower tips with wider bottoms, i.e., cone-like shaped microneedles as shown in Figure 3c. In all, the control of the microneedle shape can easily be achieved by repeating the above procedures, i.e., polymer coating and replication. In Figure S6, the change in the radius of the needle tip is plotted as a function of the number of such cycles.

The penetration test of our microneedles was carried out with porcine skin and eco-flex elastomer using differently shaped microneedles. Figure 3d,g shows photo images of the microneedle arrays that were halfway inserted into the porcine skin (intentional partial indentation), and the inset SEM images in both show the microneedle arrays that were used for the penetration test. The penetration was done by gently pressing the arrays into skin manually. Note here that the microneedle arrays in Figure 3d are in a simple cylindrical shape, while those in Figure 3g are cone like in shape, respectively. Under the application of external force to insert microneedle arrays into the skin, the shape of microneedles has a profound effect on the penetration mechanics and pain experienced by patients. First, the smaller the tip radius, the lower the penetration force that is needed for the insertion. More importantly, the fracture of skin induced by the penetrating microneedles can have quite different fracture modes depending on the tip shape of the microneedles [21,22]. Shown in Figure 3f,i are the cross-sectional OM images of the needle-penetrated porcine skins, which clearly show the different modes of skin fracture. For cylindrical microneedles, as shown in Figure 3f, the penetrated cross-section looks like a rectangular depression (refer to the delineated red-dotted line). In this case, the fracture in the porcine skin is a mode II crack, or sliding mode, where the shear stress acts parallel to the plane of the crack. The fractured skin fragments are largely remaining on the recessed region, leading to increased penetration force.

On the contrary, the penetrated cross-section shows a V-groove shape, as shown in Figure 3i, for cone-like microneedles. The fracture of the porcine skin is a mode I crack, or opening mode, where the tensile stress acts perpendicular to the plane of the crack. In this case, the fractured skin fragments are displaced sideway, leading to smaller penetration force compared to the mode II crack. Therefore, mode I crack is favorable for skin penetration due to its low penetration pressure, compared to mode II crack. It should be noted that the microneedle arrays could be fully inserted into the porcine skin to the penetration depth of ~800 μm. The difference in skin fracture modes induced by microneedles having different shapes can be easily identified from the top-view OM images of Figure 3e,h. Note here that an elastomeric eco-flex film as a skin simulant was used for the test shown in Figure 3e,h. With cylindrical microneedles, the radii of the penetration marks are almost the same as those of the microneedles, as shown in Figure 3e. However, the radii of the penetration marks are much larger than those of the microneedles for cone-shaped microneedles, as shown in Figure 3h. This is due to the laterally displaced skin fragments during penetration in mode I crack opening.

**Figure 4:**
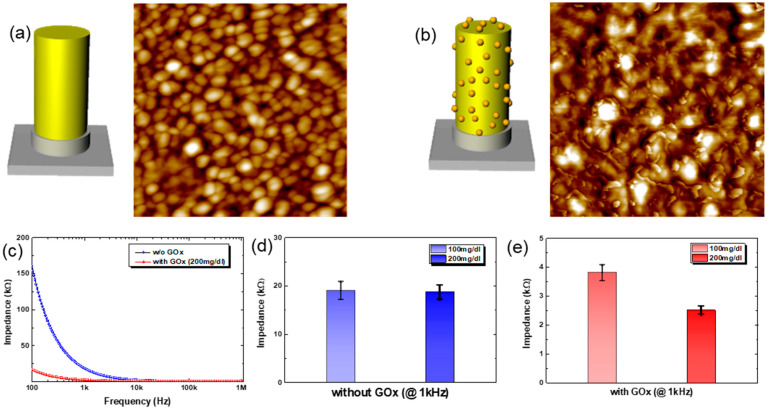
Immobilization of GOx onto Au electrode. (**a**,**b**) Schematic drawing of MEAs (**a**) with and (**b**) without GOx immobilization and the corresponding AFM images of the Au on a Si, respectively (scan size: 1 μm × 1 μm, z-scale: 12 nm). (**c**) Impedance vs. frequency plots for MEAs with or without immobilized GOx, respectively. (**d**) Impedance from MEAs without GOx, under different concentrations of glucose. (**e**) Impedance from MEAs immobilized with GOx under different concentrations of glucose solution.

The fabricated MEAs can be used for the continuous monitoring of glucose level in diabetic patients in wearable form with minimally invasive characteristics. In this preliminary work, we have used impedance spectroscopy to measure the change in glucose concentration in a continuous fashion. It should be noted that there is no need for any sensing chemistry in typical impedance spectroscopy for glucose monitoring in literature. However, this makes the method vulnerable to other variables such as temperature, moisture, or change in electrolyte balance, resulting in poor accuracy for practical application. However, here we have combined impedance spectroscopy with specific sensing chemistry for highly accurate, sensitive, and reliable glucose detection. The sensing chemistry is based on the glucose oxidase, GOx. The impedance between electrodes in MEAs, immobilized with GOx, changes according to the reaction between glucose and the GOx enzyme. The glucose-GOx reaction induces a change in impedance mainly by a change in capacitance and Warburg impedance, which makes the method reliable, quick, and sensitive under complex measurement conditions [29,30]. Shown in Figure 4a,b are the schematic drawings for electrodes and the corresponding AFM images before/after the GOx immobilization. For the AFM measurements, Au (80 nm) was deposited onto a planar Si substrate as an electrode. Before the immobilization of the GOx enzyme, as shown in Figure 4a, the electrode surface reveals the grains of Au. After the immobilization of GOx onto Au electrode, however, distinct small nanoscale features are observed on the Au electrode surface, as shown in Figure 4b. The nanoscale features are due to the immobilized GOx on the Au electrode. Note here that the MEA substrate was covered with spun-cast PDMS, except for the microneedle (3D) electrodes and contact pads, before the GOx immobilization onto MEAs for glucose sensing, to minimize the unwanted cross-talk among electrodes when in contact with liquid electrolytes (Figure S7). The gray-colored region in the schematic drawings in Figure 4a,b, therefore, denotes the coated PDMS layer to minimize cross-talk.

Figure 4c shows the plot for the change in the real part of impedance between a reference electrode (center electrode in the 3 × 3 arrays) and another electrode in the array under a fixed concentration of glucose solution (200 mg/dL). First of all, the GOx-immobilized MEAs show much lower impedance compared to that of MEAs having no GOx. This is due to the enzymatic reaction between GOx and glucose, which generates electroactive species such as hydrogen peroxide and gluconic acid [30]. These electroactive species ionize in the solution (here PBS buffer at a pH of ~7). Note here that the reactions occur near the electrodes that have immobilized GOx. This enhances ion diffusion to the electrode, reducing Warburg impedance and thus the measured impedance, too. In addition, the aggregation of the charged ionic species near the electrode decreases the capacitance by the electrical double layer, C_EDL_, which again contributes to the reduction in impedance. Also, the measured impedance decreases with the frequency in the frequency range from 100 Hz to 1 MHz, suggesting that the movement of ionic species is largely responsible for the change in impedance, such as C_EDL_ and Warburg impedance. This can be used to enhance the accuracy and reliability of the sensor by measuring at multiple frequencies. Moreover, it might be used to detect different analytes, due to the fact that different analytes show their unique frequency responses.

As for the choice of frequency, we measured the impedance at 1 kHz, and the results with or without GOx are shown in Figure 4d and Figure 4e, respectively. Without GOx immobilization, the Au-coated MEAs show no noticeable difference in impedance values with varying concentrations of glucose solution at 1 kHz, as shown in Figure 4d. With the immobilization of GOx on Au-coated MEAs, however, there is a distinct difference in the impedance values depending on the concentration of glucose solution at 1 kHz, as shown in Figure 4e. It should be noted here that the absolute values of impedance from MEAs with/without GOx immobilization are quite different: ~20 kΩ for MEAs without GOx vs. 2~4 kΩ for MEAs with GOx, i.e., an order of magnitude difference. This is due to the specific chemistry between GOx and glucose. Similar approaches show quite good detection of analytes using other receptors [31,32]. In all, we verify that our approach, impedance spectroscopy with specific sensing chemistry, works well for glucose detection.

**Figure 5:**
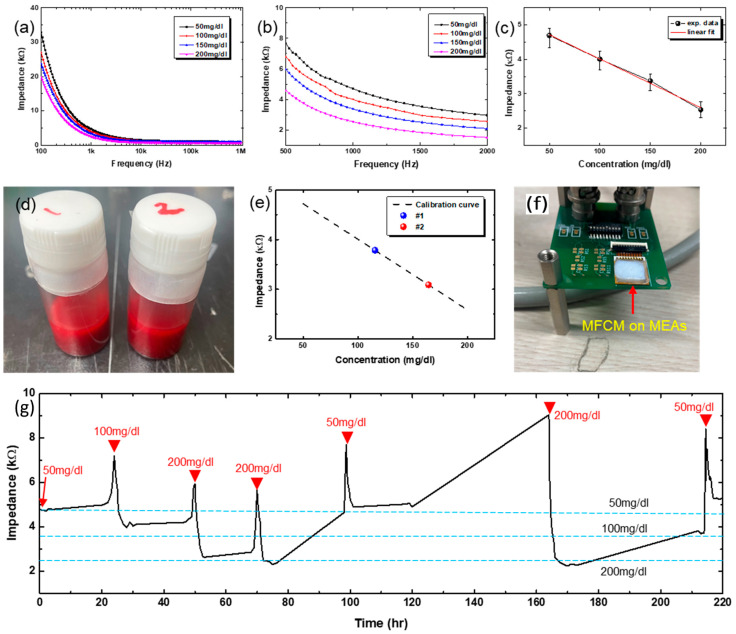
Impedance spectroscopy and continuous glucose monitoring. (**a**) Impedance of various glucose solutions with GOx-immobilized MEAs. (**b**) Magnified view of impedance spectra around 1 kHz. (**c**) Least-square fit of the experimental impedance vs. glucose concentration data. (**d**) Photo image of animal blood having different concentrations of glucose. (**e**) Measured glucose concentrations of the animal blood samples, plotted onto the calibration curve. (**f**) Photo image of measurement setup. (**g**) Long-term monitoring of glucose level by applying different concentration of glucose solution.

Figure 5a shows the change in impedance spectra for different concentrations of glucose solution, ranging from 50 mg/dL to 200 mg/dL. Shown in Figure 5b is the magnified view of Figure 5a, around 1 kHz, which shows quite a distinct difference in impedance over the frequency range from 500 Hz to 2 kHz. We used the impedance values at 1 kHz, and the corresponding values are plotted in Figure 5c as a function of glucose concentration. The impedance decreases with glucose concentration, and the change is quite linear over the investigated range of glucose concentration. The experimentally measured data are fitted into a line by linear regression, shown as a red line, and the fitted line plays the role of a calibration curve.

Figure 5d shows the photo image of animal blood samples having different concentrations of glucose, denoted as #1 and #2. Using these blood solutions, the impedance values at 1 kHz were measured and plotted in Figure 5e, indicated by #1 and #2. The calibration curve in Figure 5c was re-drawn for comparison purposes. As shown, the measured impedance values with animal bloods having pre-defined concentrations of glucose lie on the calibration curve with negligible errors. From this, glucose concentration was determined to be 109~123 mg/dL for the #1 sample and 153~176 mg/dL for the #2 sample, respectively. The same blood samples were measured by a commercial glucose sensor and yielded 111 mg/dL for the #1 sample and 159 mg/dL for the #2 sample, respectively. The glucose concentrations measured by our GOx-immobilized MEAs are comparable to the value measured by the commercial sensor. Note here that the impedance (thus glucose concentration) measurement with our MEAs was carried out with all 8 electrodes (except a reference electrode), yielding slightly different values of glucose concentration, which is the reason for the ranged values of glucose concentration instead of a single value (Figure S8). These data were all tabulated in Table 1.

To check the possibility of the long-term glucose measurements, the MEAs were bonded to a printed circuit board having a comprehensive circuit for impedance spectroscopy, as shown in the photo image of Figure 5f. The MEAs were inserted into the micropig Franz cell membrane (MFCM) that was pre-soaked with a specific concentration of glucose solution for the measurement. As the MFCM dries with time, the impedance starts to increase. Once the impedance starts to increase by MFCM drying, the MEAs with MFCM are stored in a Petri dish that is saturated with water vapor to re-hydrate the MFCM. To re-start the measurements, glucose solutions having different concentrations were dropped onto the MFCM. Shown in Figure 5g is the long-term test result, plotted impedance values at 1 kHz as a function of time. As shown, the GOx-immobilized MEAs were found to be able to measure the glucose level for ~10 days. The blue-dotted horizontal lines denote the impedance values corresponding to different concentrations of glucose as noted, based on the calibration curve shown in Figure 5c. The slight discrepancy between long-term measurement results and the calibrated values is believed to be due to the uncertainty in the glucose concentration. As explained above, we have added solution drops onto the MFCM intermittently (indicated by red arrowheads in Figure 5g), which may mix with the remaining glucose solution inside the MFCM, making the exact determination of glucose concentration difficult. Another possible cause is that the MFCM seems to partially dry even with storage in a water-saturated environment. Once dried, the MFCM cannot absorb the glucose solution as before, meaning a kind of irreversible permanent degradation. This may lead to underestimation of glucose level, because some amount of glucose may be trapped in the inactive region of MFCM. Nevertheless, the GOx-immobilized MEAs have shown the possibility for long-term stable operation, which is critically important for CGM.

**Figure 6:**
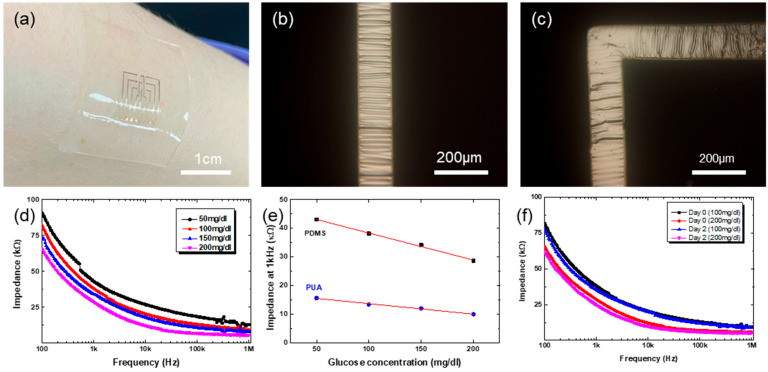
Stretchable MEAs. (**a**) Skin-attached MEAs made of elastomeric PDMS. (**b**,**c**) are the OM images of wrinkled Ti/Au interconnection lines, straight and right-angled lines for (**b**,**c**), respectively. (**d**) Impedance spectra with different concentrations of glucose solution. (**e**) Comparison of impedance between polymeric and elastomeric MEAs at the same glucose concentrations. (**f**) Repeatability test of stretchable MEAs.

The MEAs are made of flexible polymer, PUA. The same process steps shown in Figure 1a can be used to fabricate stretchable MEAs with elastomeric material such as PDMS. On the Au/Ti patterned hole-arrayed PDMS stamp, pouring and thermal curing of elastomeric PDMS, instead of UV-curable PUA prepolymer, followed by peeling off the MEAs, complete the fabrication of stretchable MEAs. While the flexible polymeric MEAs can be deformed into cylindrical shapes only, the stretchable elastomeric MEAs can be conformed onto complex curvilinear surfaces. Further, the elastomeric nature enables soft contact with biological materials, which may be beneficial for contacting very soft materials such as brain tissues. Shown in Figure 6a is the conformable nature of elastomeric MEAs made of PDMS. It conforms and attaches quite firmly onto the human skin surface.

It should be noted that the metallic materials, Au and Ti in this work, should also be made stretchable for the fabrication of stretchable MEAs (MNAs made of PDMS are intrinsically stretchable). For this, the well-known mechanical wrinkling of metals was employed here [33,34,35]. During the patterned metal evaporation with a shadow mask, the PDMS stamp was pre-stretched to 5% biaxially. Upon release of the pre-strain after the deposition, the metallic lines become buckled, as shown in Figure 6b,c. There are interconnection lines having both straight and right-angled parts. If the metallic lines are straight in one direction only, a uniaxial stretching during metal evaporation would be enough for the wrinkling. For lines having right-angled parts, however, biaxial pre-strain should be applied for the buckling along the whole lines, as shown in Figure 6c. The buckled metallic lines are reversibly stretchable: they flatten upon stretching strain while recovering their buckled state upon the release of external stretching strain. Overall, applying biaxial pre-strain to the PDMS stamp during patterned metal deposition leads to the mechanical buckling of interconnection metal lines, enabling the stretchable MEAs. It should be noted that the needles do not experience enough strain for buckling by such small biaxial pre-strain, because the applied biaxial pre-strain is largely absorbed by the inter-needle planar part of the MEAs. Thus, the metallic layer on 3D microneedles remains intact, not buckled at all. If the uniaxial pre-strain is large enough, 30%, for example, then the needle surface becomes buckled (Figure S9). Such large biaxial pre-strain could not be applied due to limited availability of proper equipment.

The fabricated stretchable MEAs work well for the impedance measurements (Figure S10), too, as shown in Figure 6d. For the impedance measurement, GOx was immobilized onto the Au surface on stretchable MEAs. The impedance spectra with stretchable MEAs show quite similar behavior to those with polymeric MEAs shown in Figure 5a. With the concentration of glucose solution, the impedance values decrease. While the impedance spectra look similar, there are differences in the absolute values of the impedance, as shown in Figure 6e. The impedance values are smaller with polymeric MEAs than with elastomeric MEAs. The exact reason is unclear yet, but the micro-cracks in the Au electrode are likely to induce higher impedance in elastomeric MEAs. Regardless of the difference in the absolute values of impedance, the elastomeric MEAs can still be successfully used for the glucose sensing. Shown in Figure 6f are the impedance spectra for different concentrations of glucose solution with stretchable MEAs. The measurement here was done by dropping glucose solution onto the stretchable MEAs. As shown, the stretchable MEAs show reliable impedance spectra with different glucose concentrations. In all, the unique fabrication strategy developed in this work enables the fabrication of flexible and even stretchable MEAs, and both the MEAs have successfully been applied for the monitoring of glucose level based on impedance spectroscopy.

## 4. Conclusions

In summary, the MEAs immobilized with GOx have been demonstrated for the successful monitoring of glucose level by impedance spectroscopy. The MEAs have been fabricated using a simple and low-cost polymeric replication method by combining the MNA fabrication and patterned electrode in a single step. The formation of 2D/3D patterned electrodes was prepared by shadow evaporation, where the transparent and conformable shadow mask was also fabricated by a modified replica molding technique. Those unique properties of the shadow mask have enabled manual alignment onto a stamp, which completely removes the costly photolithography. The GOx-immobilized MEAs have shown systematically different impedance spectra with different concentrations of glucose solution thanks to the specific chemistry between GOx and glucose. This has enabled fast and accurate determination of glucose level. Further, the same processes could be used for the fabrication of stretchable elastomeric MEAs, where the stiff metallic electrodes were mechanically buckled into a sinusoidally wavy shape for stretchability. The results shown in this work can find a variety of applications of flexible/stretchable MEAs for personal health monitoring and bio-signal monitoring in a continuous and wearable fashion.

## Figures and Tables

**Table 1:** Comparison of measured glucose concentration.

Blood Number	Prepared Concentration	Commercial Glucose Meter	This Work (Average)
#1 (mg/dL)	120	111	109~123 (115)
#2 (mg/dL)	170	159	153~176 (164)

## Data Availability

Data are contained within the article.

## Supplementary Materials

The following supporting information can be downloaded at: https://www.mdpi.com/article/10.3390/bios15090576/s1, Figure S1: Fabrication of PDMS stamp; Figure S2: Importance of delicate adhesion control; Figure S3: Needle shape dependency on the transfer molding; Figure S4: Vertical sidewall of polymeric shadow mask; Figure S5: Schematic drawings for the needle shape control; Figure S6: Microneedle shape control; Figure S7: Comparison of impedance from all 8 electrodes (1 electrode was set to reference one); Figure S8: Buckling of metal layer on microneedle surface under large uniaxial pre-strain; Figure S9: Passivation of MEAs with PDMS; Figure S10: Electrical connection for the stretchable MEAs.

## Abbreviations

The following abbreviations are used in this manuscript:

CGM	Continuous glucose monitoring
GOx	Glucose oxidase
MEAs	Microneedle electrode arrays
MNAs	Microneedle arrays
ECG	Electrocardiography
EMG	Electromyography
EEG	Electroencephalography
PDMS	Polydimethylsiloxane
NHS	N-hydroxysuccinimide
MPA	3-Mercaptopropionic acid
EDC	1-ethyl-3-(3-dimethylaminopropyl)carbodiimide hydrochloride
PBS	Phosphate buffer solution
PUA	Polyurethane acrylate
PI	Polyimide
SAM	Self-assembled monolayer
MFCM	Micropig Franz cell membrane
FPCB	Flexible printed circuit board
