# Supplementary material for: Flexible and Stretchable Microneedle Electrode Arrays by Soft Lithography for Continuous Monitoring of Glucose
Source: Biosensors (Basel). 2025 Sep 2;15(9):576. doi: 10.3390/bios15090576 (PMC12467386; doi:10.3390/bios15090576)
Supplement: Supplementary file 1 [file biosensors-15-00576-s001.zip › biosensors-3808780-supplementary.pdf]

Supporting Information

Flexible and Stretchable Microneedle Electrode Arrays by Soft Lithography for Continuous Monitoring of Glucose

Yong-Ho Choi †, Honglin Piao †, Jia Lee, Jaehyun Kim, Heon-Jin Choi * and Dahl-Young Khang *

Department of Materials Science and Engineering, Yonsei University, Seoul 03722, Republic of Korea; yhyhyh825@naver.com (Y.-H.C.); hongrim@yonsei.ac.kr (H.P.); jia.lee@yonsei.ac.kr (J.L.); kimjae5020@yonsei.ac.kr (J.K.)

* Correspondence: dykhang@yonsei.ac.kr (D.-Y.K.), hjc@yonsei.ac.kr (H.-J.C.)

† These authors contributed equally to this work.

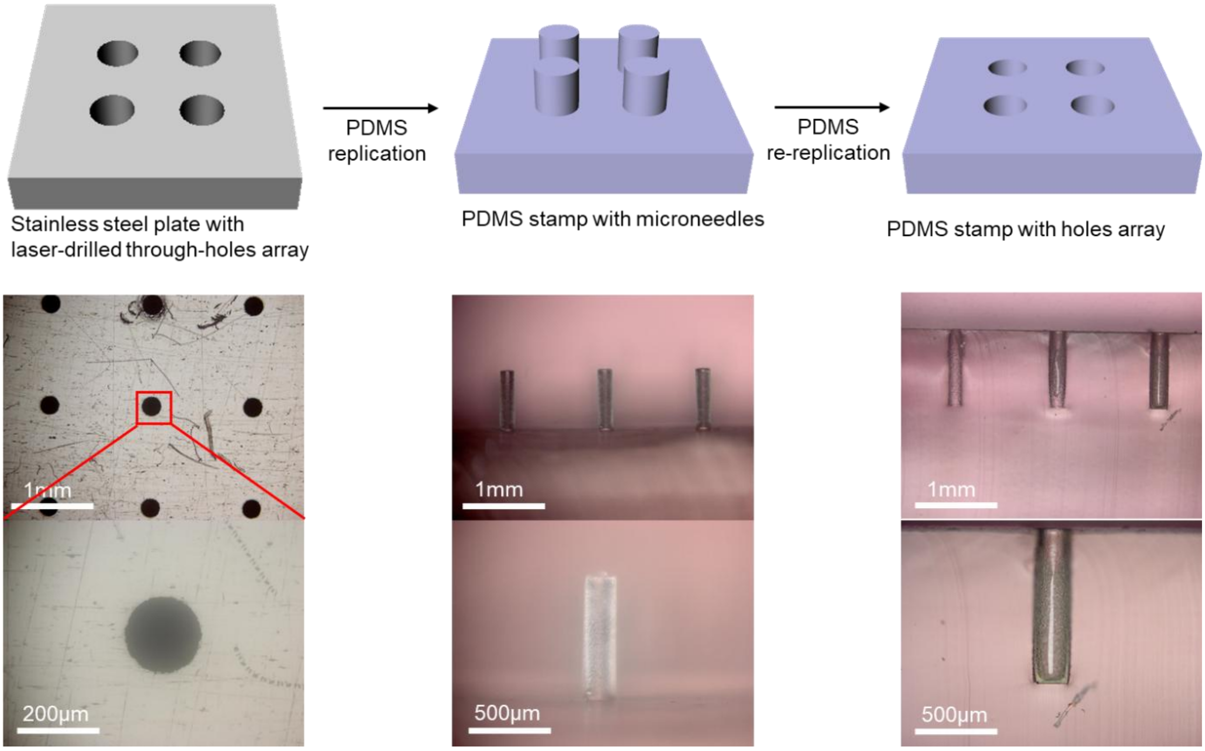

Figure S1. Fabrication of PDMS stamp. (top) Schematic illustration of PDMS stamp fabrication by replica molding, (middle and bottom) OM images of samples at each stage. A stainless steel plate (~0.8mm thick; left column) was laser-drilled and polished, followed by replication of PDMS stamp with the holed-plate as a master. Here the replicated PDMS stamp has protruding needle structures (middle column). After oxygen plasma treatment followed by F-SAM treatment, another round of replica molding was carried out to fabricate PDMS stamp with recessed holes (right column), using needle-shaped PDMS stamp as a mold. Cross-sectional OM images were shown in middle/right columns, which clearly denotes the protruding/recessing pattern features.

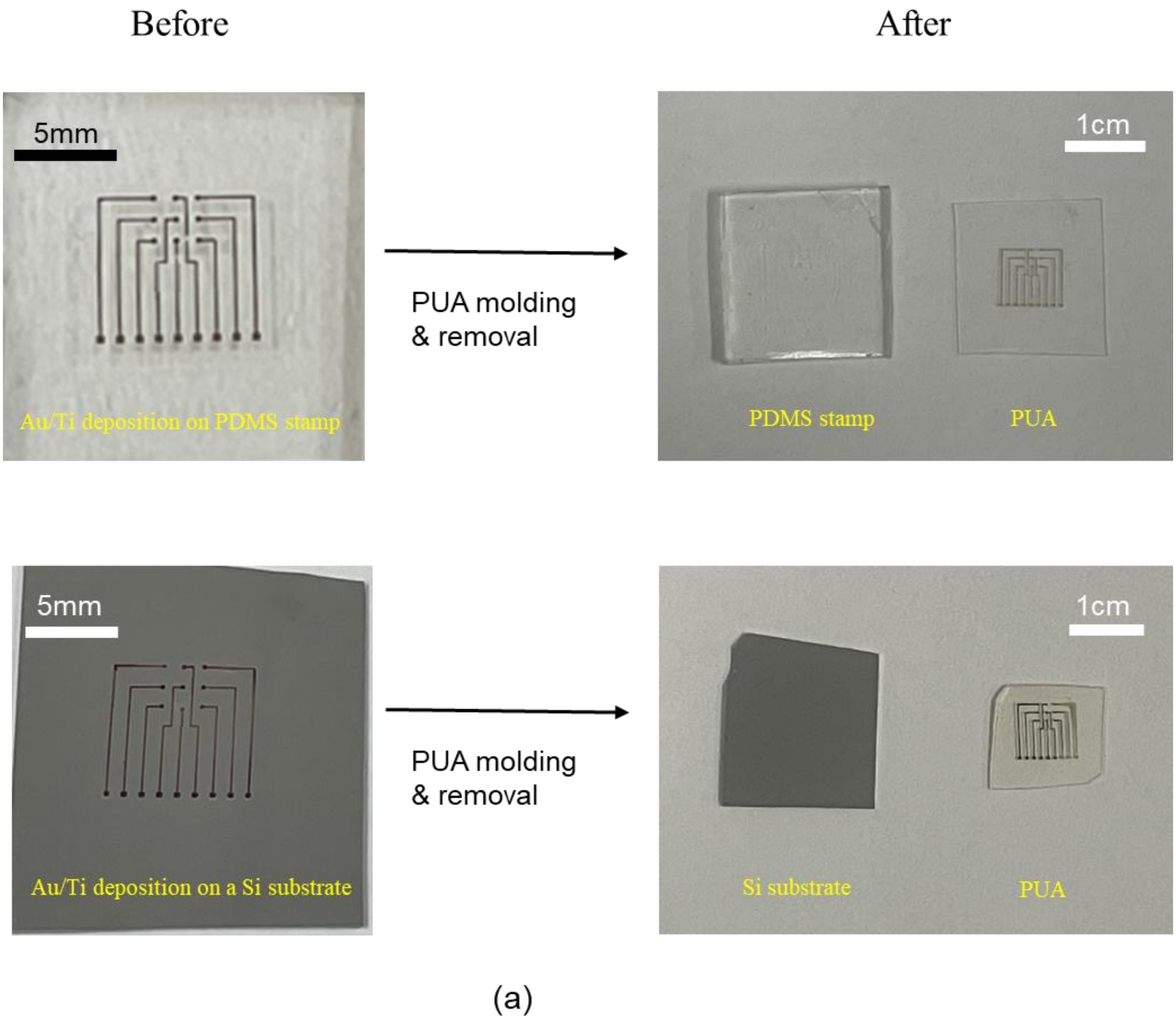

Figure S2. Importance of delicate adhesion control. (a) Photos of Au/Ti metallized planar PDMS (top) and Si (bottom), respectively. This combination, i.e., Au deposition followed by Ti deposition, has led to the successful transfer of patterned metals onto PUA. After transfer molding, it can be clearly seen that the original deposition substrates (PDMS, Si) look quite clean. In the meanwhile, the molded PUA takes the whole pattern of metal lines. This is due the weak adhesion between Au and substrates (Si or PDMS) and good adhesion between Ti and PUA.

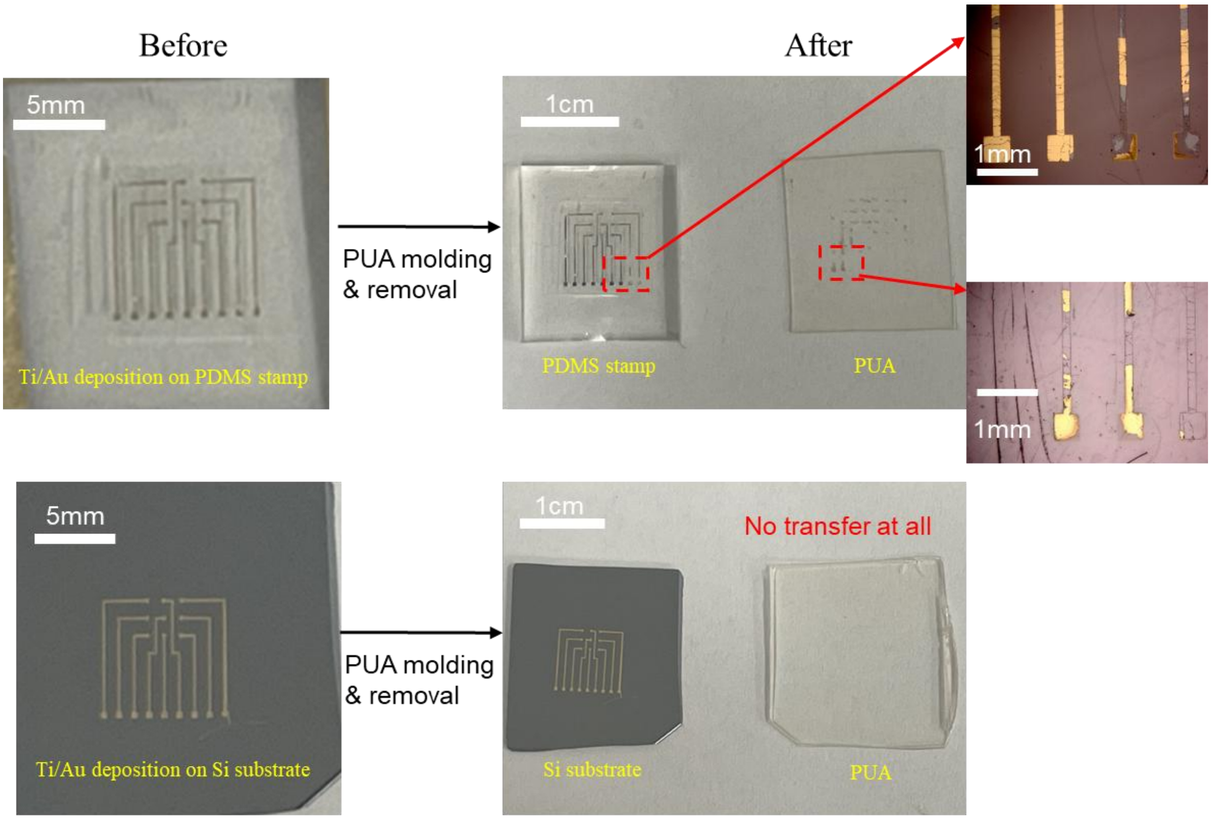

(b)

Figure S2. (continued) (b) Photo and OM images showing unsuccessful transfer of metal patterns, formed by Ti/Au metallization on PDMS (top) and Si (bottom). Note here that the order of metallization is reversed, from Au/Ti to Ti/Au. The Ti layer has good adhesion with PDMS and Si, while the overlying Au does not have enough adhesion with the molding PUA. This has led to unsuccessful transfer of the metallized patterns onto PUA. Also, even with Ti layer, the intrinsically low energy surface of PDMS yielded partial transfer (shown OM images on top right).

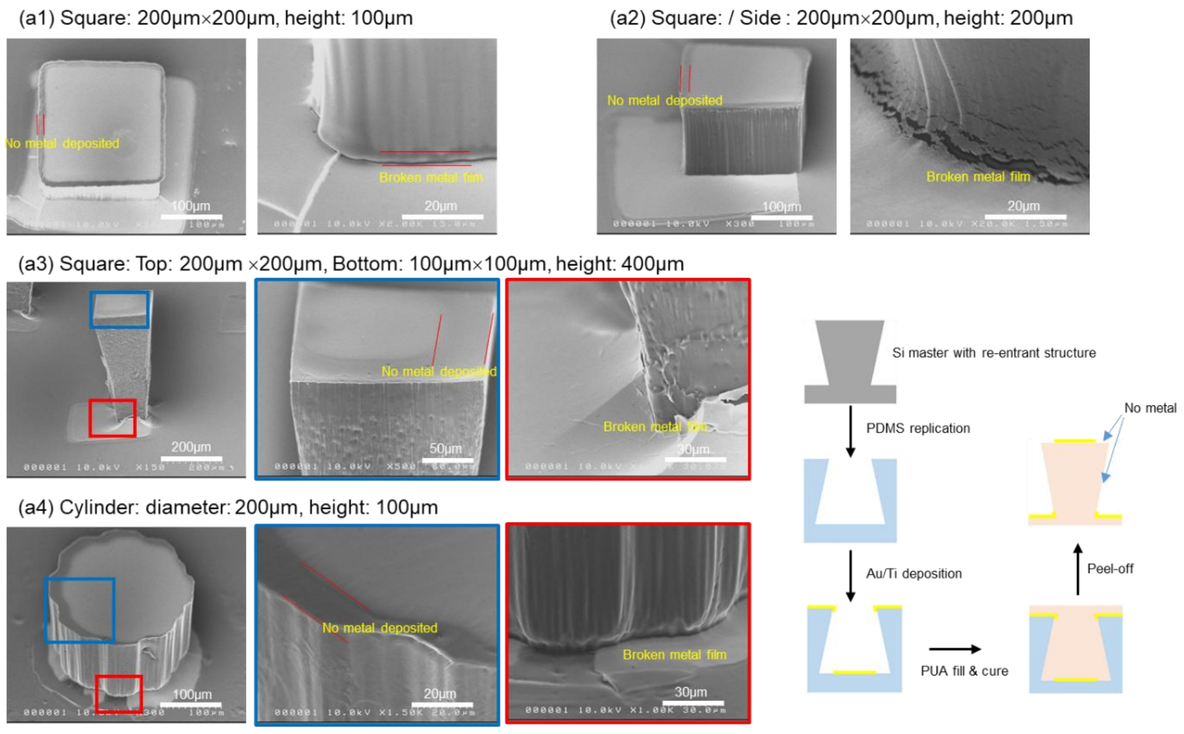
(a1) Square: 200μm×200μm, height: 100μm
(a2) Square: / Side : 200μm×200μm, height: 200μm
No metal deposited
Broken metal film
100μm
20μm
(a3) Square: Top: 200μm ×200μm, Bottom: 100μm×100μm, height: 400μm
200μm
50μm
30μm
(a4) Cylinder: diameter: 200μm, height: 100μm
Si master with re-entrant structure
PDMS replication
No metal
Au/Ti deposition
Peel-off
PUA fill & cure

(a)

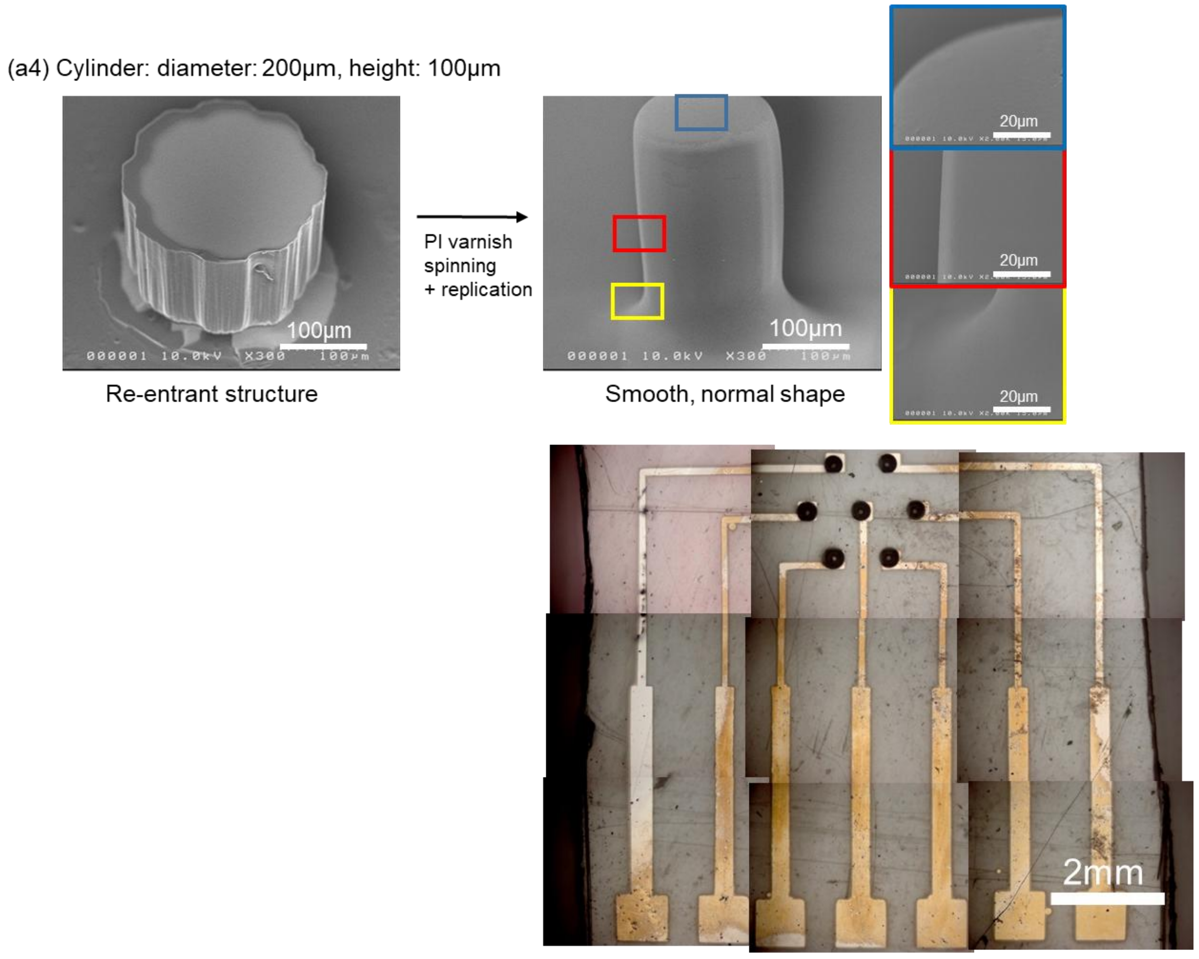
(a4) Cylinder: diameter: 200μm, height: 100μm
PI varnish
spinning
+ replication
100μm
20μm
Re-entrant structure
Smooth, normal shape
2mm

(b1)

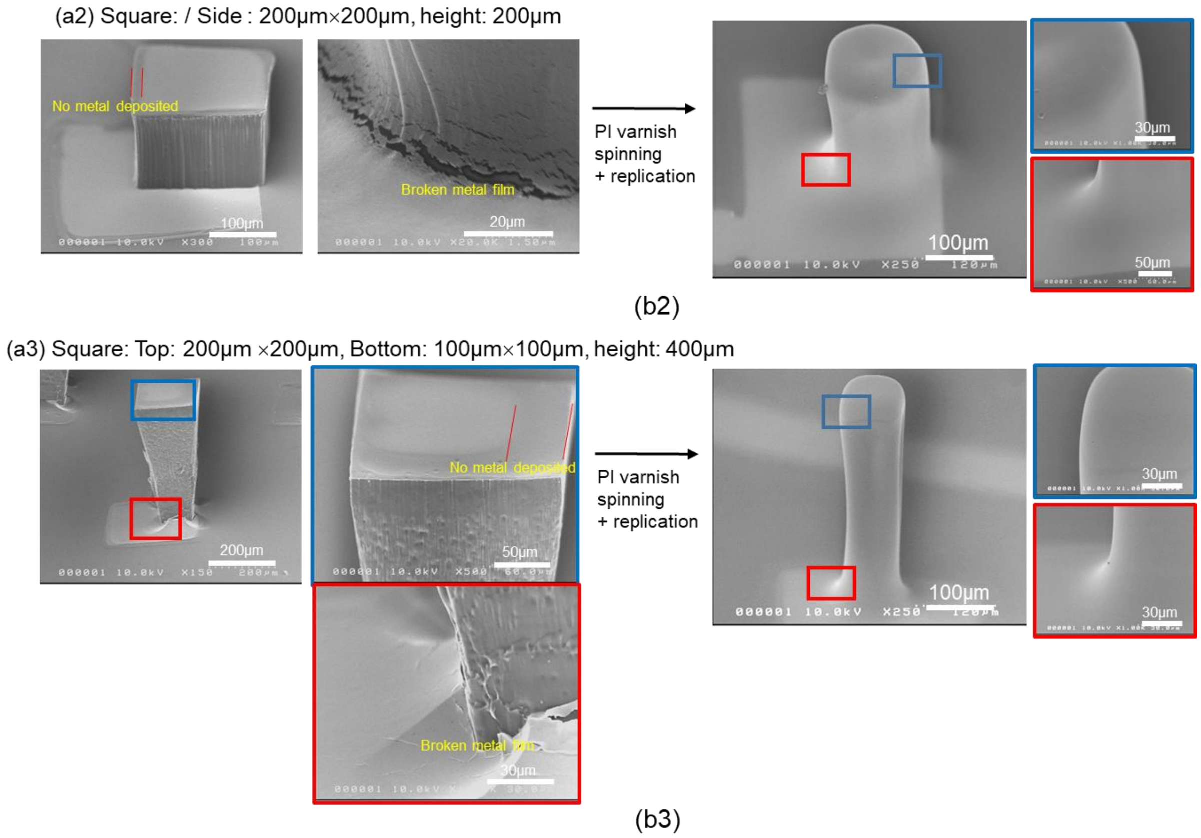

Figure S3. Needle shape dependency on the transfer molding. (a) The transfer molding was unsuccessful when the needle shape takes the re-entrant one, that is, wider top with narrower bottom. SEM images shown in (a1), (a2), (a3) and (a4) are the low-magnification images showing the metal deposition on top surface together with expanded view of needle bottom. As shown, due to re-entrant needle structure, top surface coverages of metal were not complete, nor the electrical connection of metal lines at the needle bottom region. Shown in bottom right is the schematic drawing for the incomplete top surface metal coverage and the broken metal interconnection at the needle bottom. (b) The re-entrant shaped needles can be modified into a normal (narrower top with wider bottom) shape by polymer coating, and this modified needles can be successfully used for the transfer molding. (b1) SEM and OM images of needle structure before (re-entrant; shown in a4) and after the polymer coating. The coated polymer converts the shape of needles from re-entrant to normal ones, which has enabled seemless replication molding metal lines with needle shape altogether. Shown in bottom right is the collection of OM images into the whole shape of the sample, including contact pads, metal interconnections to each needles (9 needles in total), and needles, showing the successful replication molding. (b2, b3) SEM images of re-entrant structured needles and modified ones by polymer coating, highlighting the successful replica molding of microneedle electrodes, quite similar to (b1).

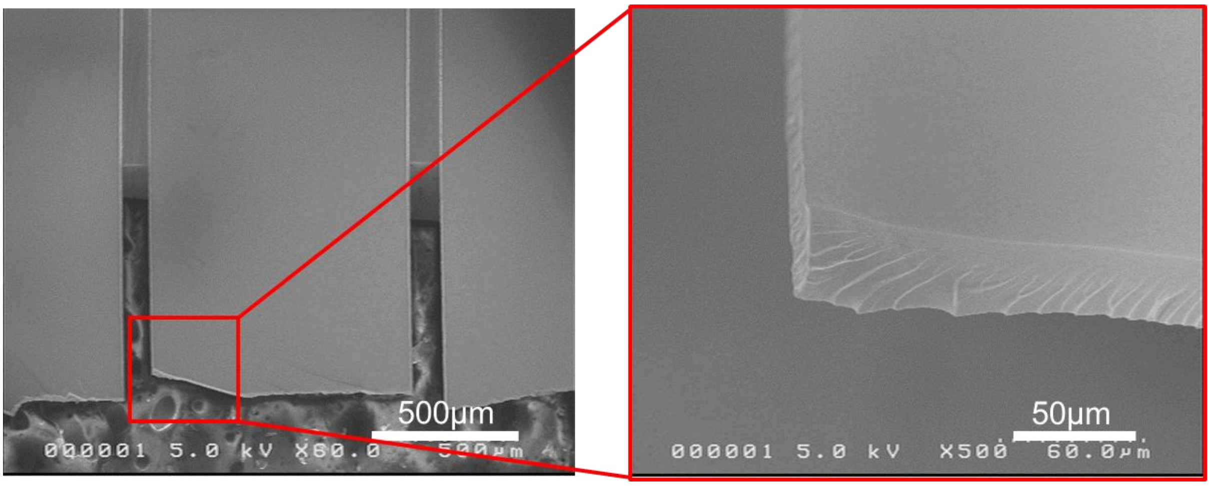

Figure S4. Vertical sidewall of polymeric shadow mask. Cross-view of polymeric shadow mask fabricated by the modified replica molding (left), and its expanded view (right). This upright sidewall of polymeric shadow mask, together with conformable flexible nature, enables high fidelity pattern transfer as shown in the Fig. 2 of Main Text.

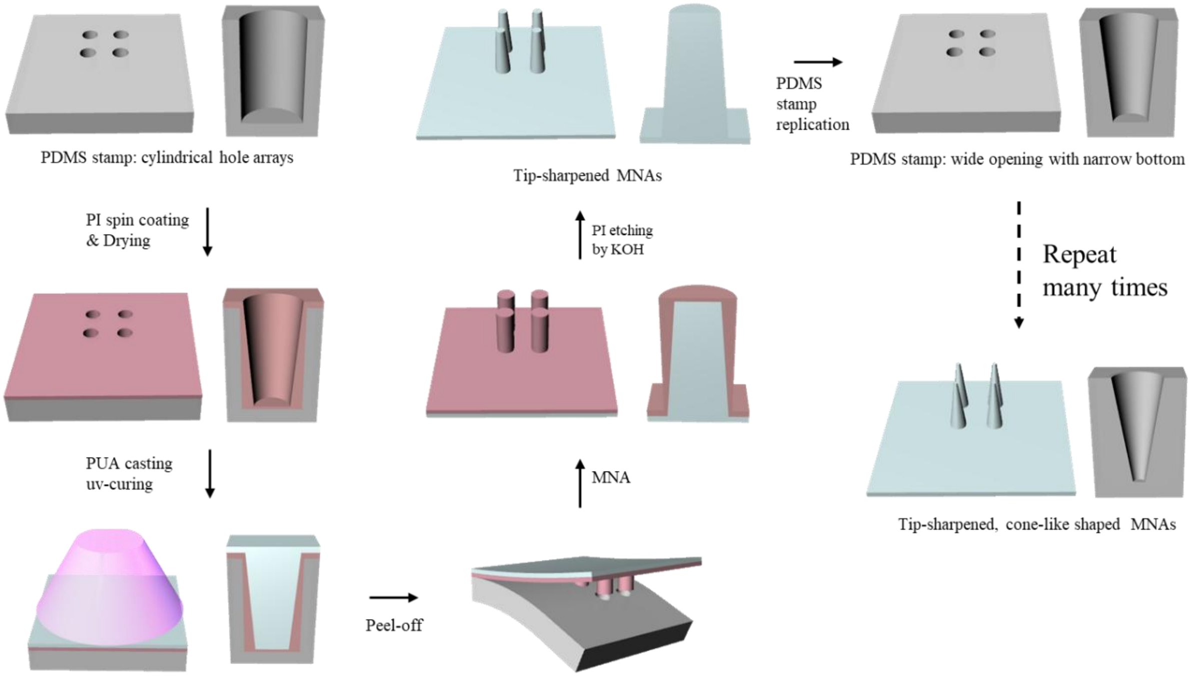

Figure S5. Schematic drawings for the microneedle shape control. The spin-coating of viscous polymeric liquid, PI varnish in this work, makes the non-uniform coating, thick layer on bottom while thin on top regions. The non-uniformly coated hole-arrayed PDMS stamp can be used to fabricate sloped microneedles by KOH etching of the PI layer. The slope and the radius of needle tip can be controllably modified by repeated application of the non-uniform coating, replication, and PI etch removal.

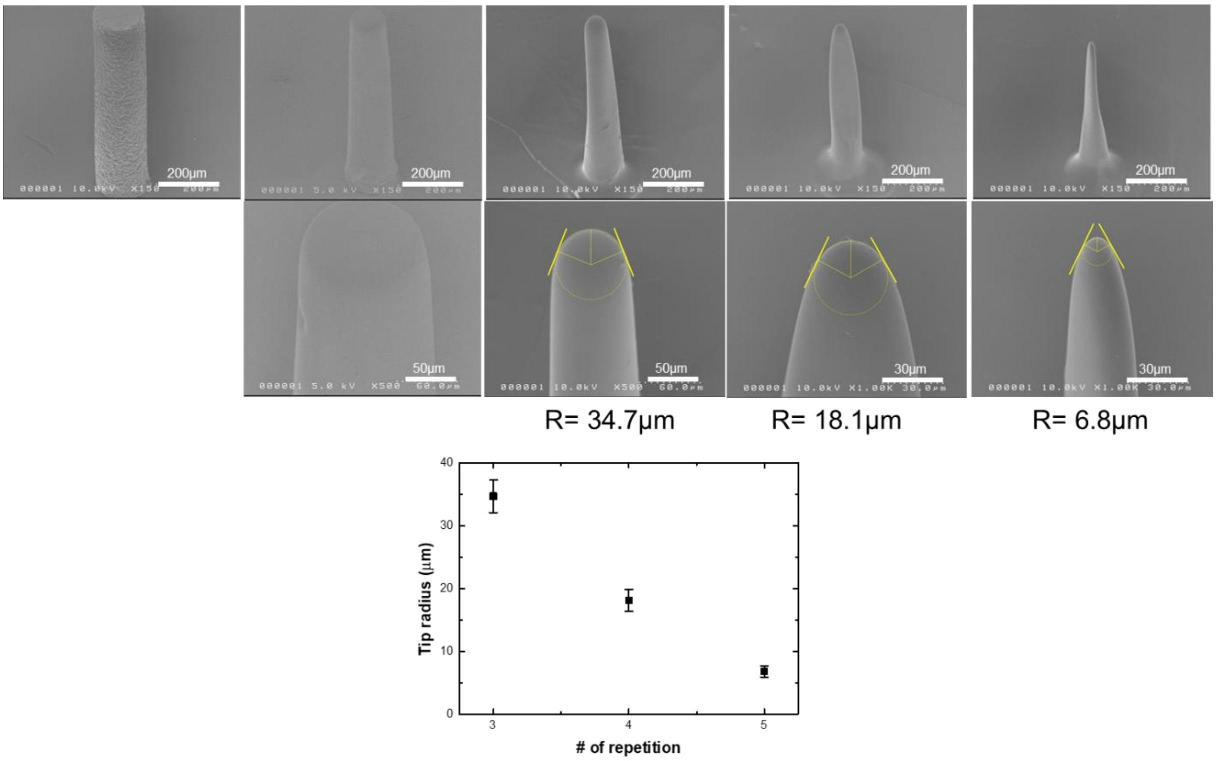

Figure S6. Microneedle shape control. (top) SEM images of microneedles, from simple cylinder (left-most) to cone-shaped (right-most) needles by repetition of the process shown in Fig. S5. (bottom) Plot for the tip radius of microneedle upon the repeated application of the processes shown in Fig. S5.

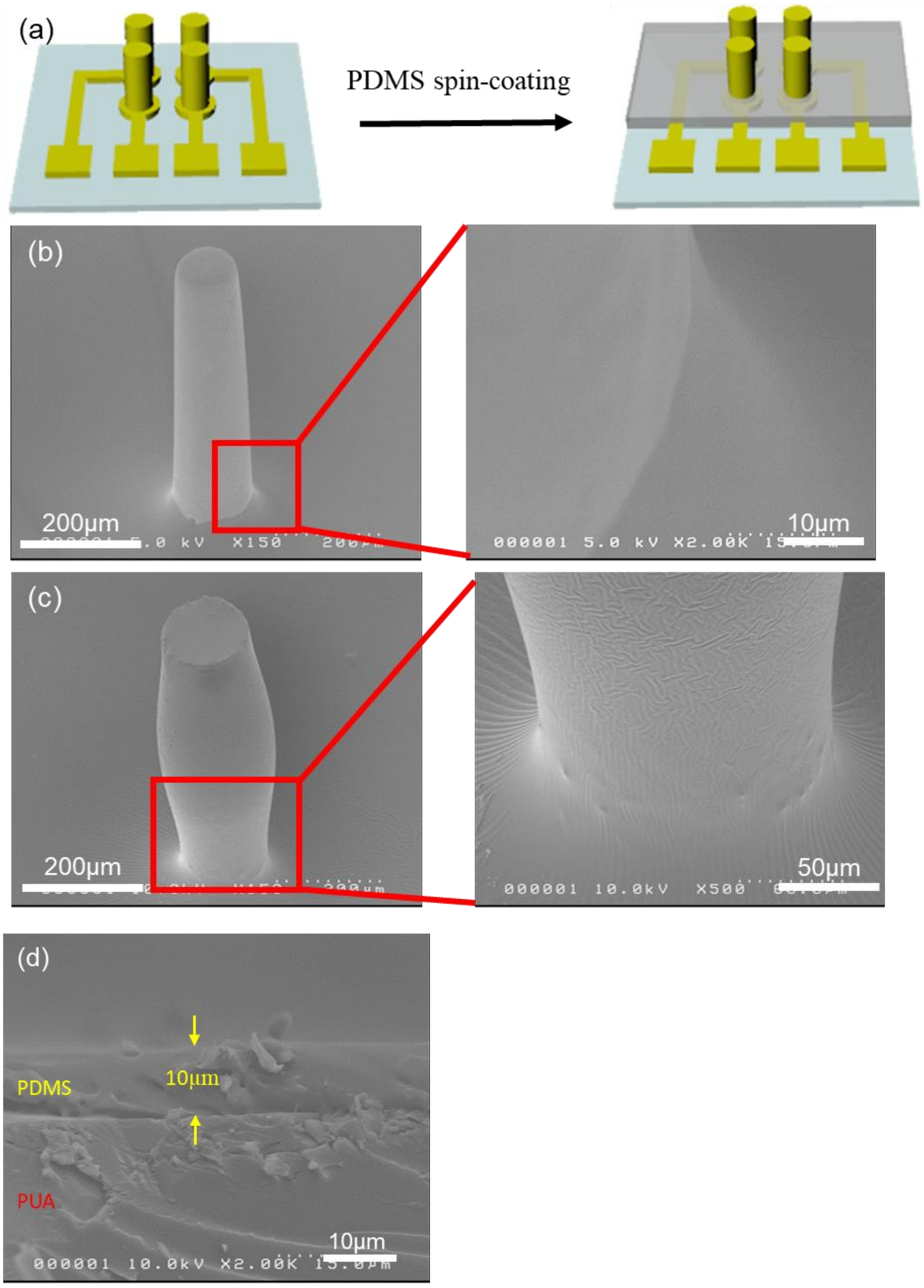

Figure S7. Passivation of MEAs with PDMS. (a) Schematic drawing for MEAs passivation with PDMS. Note here that the passivation was done on MEAs except the protruding 3D electrodes and the contact pads. (b) SEM Images of properly passivated MEAs sample; the PDMS coating does not cover the 3D electrode. The exposed Au on the 3D electrodes can be used to immobilize GOx on their surface. (c) SEM images of improper coating of PDMS onto 3D electrode surface. This unwanted coverage of PDMS onto 3D electrodes hinder the following GOx immobilization on Au surface. It is important to control the dispensed PDMS droplet volume to get proper passivation. (d) Cross-sectional SEM image of PDMS passivation layer. It can be shown that the thickness of the PDMS passivation layer is ~10μm.

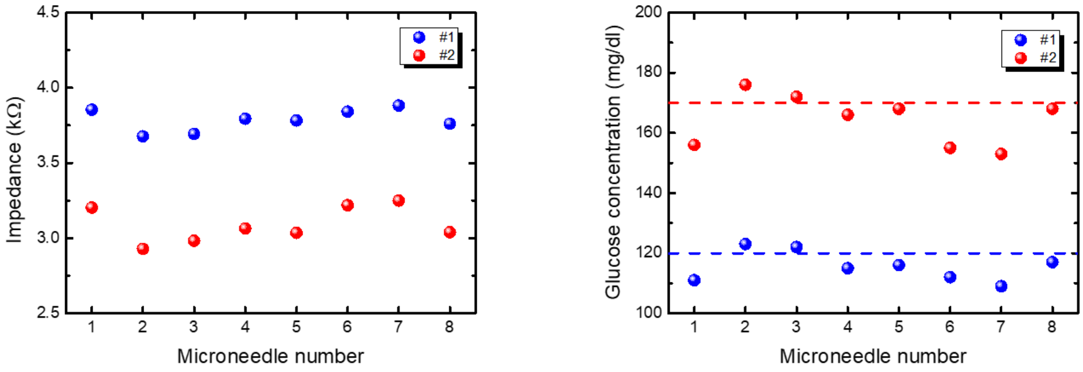

Figure S8. Comparison of impedance from all 8 electrodes (1 electrode was set to reference one). (left) Plot for the measured impedance values at 1kHz, and (right) plot for the glucose concentration determined using the calibration curve shown in Fig. 5(c) in the Main Text. The two glucose samples were at 120 mg/dl (#1) and at 170 mg/dl (#2), respectively, and are given as dashed horizontal lines in the plot shown in the right.

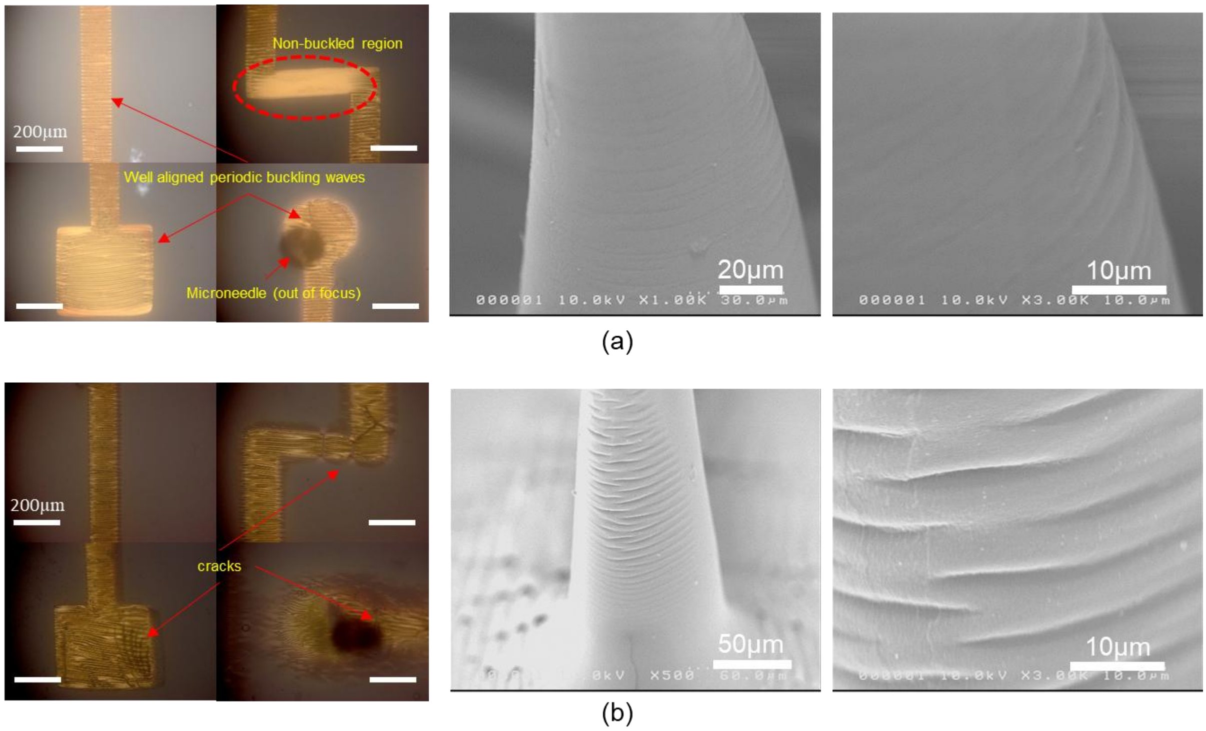

Figure S9. Buckling of metal layer on microneedle surface under large uniaxial pre-strain. To visualize the buckling of metal film on 3-dimensional microneedle surface, large uniaxial pre-strain was applied and the samples were imaged. (a) OM (left) and SEM (right) images of sample surface under the uniaxial pre-strain of 15%. Note the line-patterned metallic electrode does not buckle at all when the line is parallel to the pre-stretching direction. (b) OM (left) and SEM (right) images of sample surface under the uniaxial pre-strain of 30%. Here, there form a lot of cracks due to excessive tensile strain. In both cases, the 3-D needle surface shows clear buckling patterns, which enables the fully stretchable MEAs. If one can applied such large bi-axial pre-strain, then the whole MEAs, including both 2D interconnections and 3D microneedles would be buckled and thus be stretchable.

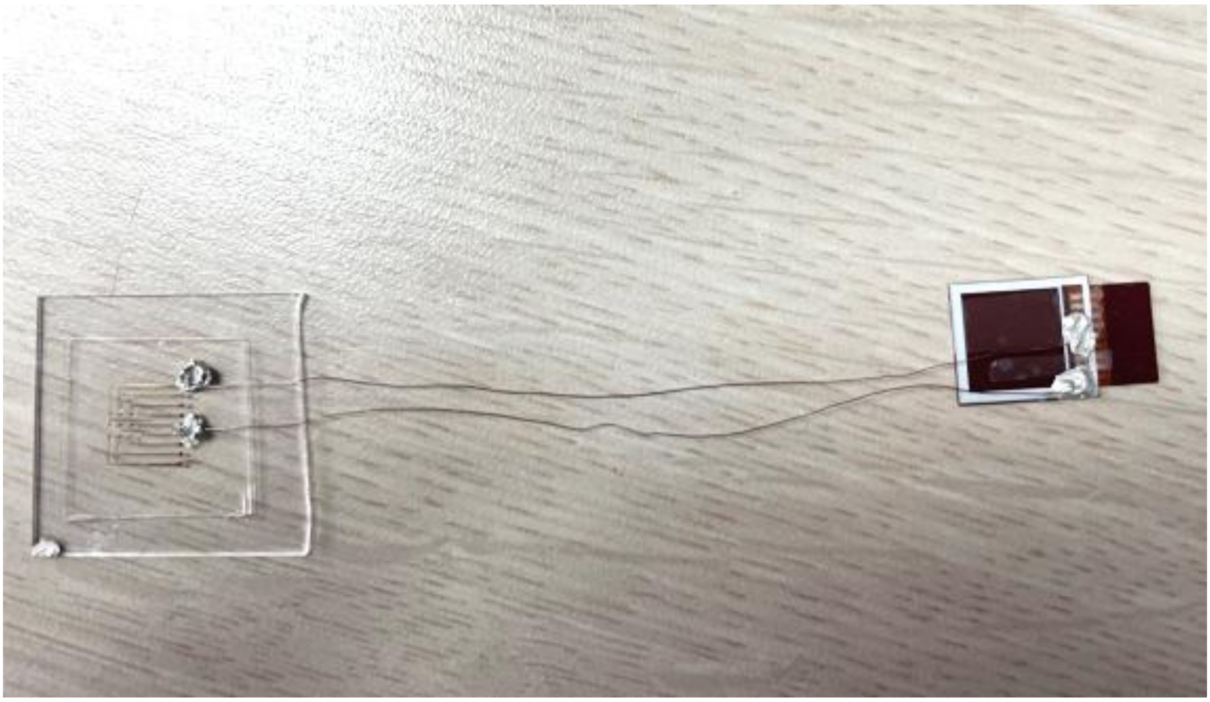

Figure S10. Electrical connection for the stretchable MEAs. The photo image shows the electrical connection between stretchable MEAs (left) and PCB (right). For the connection, a thin Au wire was carefully placed on a contact pad on the stretchable MEAs, and then covered with dropped Ag paste. The dispensed Ag paste was dried at room temperature overnight (applying temperature to dry the paste inevitably induces thermal strain in the stretchable MEAs, leading to total fracture of metal layer on it). Similar processing was done on the PCB side, i.e., Au wire contact with Ag paste drop and drying.
